# Moderating effects of smoking and drinking on the relationship between biological rhythm and psychological health and gender differences among adolescents

**DOI:** 10.1186/s12888-023-05253-2

**Published:** 2023-10-10

**Authors:** Jiaojiao Wang, Yang Xie, Huiqiong Xu, Yuhui Wan, Fangbiao Tao

**Affiliations:** 1 Department of Maternal, Child & Adolescent Health, School of Public Health, Anhui Medical University, Hefei, Anhui China; 2 Anhui Provincial Key Laboratory of Population Health & Aristogenics, Anhui Medical University, Hefei, Anhui China; 3 Moe Key Laboratory of Population Health Across Life Cycle, Anhui Medical University, Hefei, Anhui China; 4 NHC Key Laboratory of Study on Abnormal Gametes and Reproductive Tract, Hefei, Anhui China

**Keywords:** Biological rhythm, Psychological health, Smoking, Drinking, Moderating variable, Adolescents

## Abstract

**Objectives:**

To determine whether smoking and drinking moderate the correlation between biological rhythm and mental health and the role of gender differences in these moderating effects.

**Methods:**

Adolescents from three cities, all twelve middle schools (N = 7,986), named Shenzhen, Nanchang and Shenyang in China, were asked to complete a standardized questionnaire including the details of biological rhythm, psychological health, and the status of smoking and drinking. The PROCESS program was used to analyze whether smoking and drinking moderated the relationship between biological rhythm and psychological health.

**Results:**

The analyses revealed poorer psychological health and greater likelihood of smoking and drinking in participants with higher scores for biological rhythm disorder (*P* < 0.001). Specifically, smoking and drinking accelerated the relationship between biological rhythm and psychological health in the total sample (*B* = 0.05, *P* < 0.05; *B* = 0.06, *P* < 0.001) and only the subgroup of girls (*B* = 0.09, *P* < 0.05; *B* = 0.12, *P* < 0.001), respectively.

**Conclusions:**

As the findings suggest, attention should be given to smoking, drinking and gender-specific approaches employed to alleviate the psychological disorders of adolescents with biological rhythm disorders.

**Supplementary Information:**

The online version contains supplementary material available at 10.1186/s12888-023-05253-2.

## Introduction

Psychological disorders have been a global epidemic that affects the population all over the world [1]. The contribution to the global burden of the age-standardized disability-adjusted life years, owing to mental disorders, reportedly accounted for 4.89% of the burden [2]. Compared to normal individuals, people with serious psychological health problems may have a shorter life expectancy [3]. In recent years, psychological health among adolescents has gradually attracted global attention. Adolescence, a sign of developmental vulnerability, is a crucial period of psychological health development [4] and is the age group with the highest risk of suffering from psychological health disorders [5]. A cross-sectional study targeting 15,055 Chinese high school students found that more than half of adolescents suffered from emotional disturbance and anxiety; additionally, students with depression, hostility, and psychological imbalance accounted for 40.9%, 33.9%, and 30.4%, respectively [6]. A recent cross-temporal meta-analysis based on data from 1989 to 2018 reported that depression and anxiety levels were increased in Chinese adolescents [7]. Psychological health problems during adolescence are of concern, affecting the normal development and maturation of the brain, physical health [3], life satisfaction, during this period and persisting into later adulthood [8]. Additionally, adolescents with psychological disorders are vulnerable to social injustice, such as violation of human rights, social exclusion and discrimination [4]. There are many factors underlying psychological health that place adolescents in danger of psychological health problems. Consequently, recognizing these factors plays a vital role in preventing psychological disorders.

Biological rhythm is defined as biological changes with repeated daily and regular cycles, comprising hormone secretion, the sleep-wake cycle, body temperature, regular eating habits, etc. [9]. A growing number of studies have reported the relationship between biological rhythm disorders and psychological health problems in adolescents. A sample of 181 adults found that compared with healthy individuals, patients with depression showed a greater degree of disorder in biological rhythm [10]. A cross-sectional, youth-based investigation found a correlation between biological rhythm disorder and mood disorders, most notably depicted in bipolar disorder and depression; additionally, individuals who presented with an evening preference were associated with higher biological rhythm disorder [11]. Recent findings have suggested that irregular sleep increases the risk of emotional and behavioral symptoms and may predict mental health outcomes in adolescents [12]. In addition, unhealthy diet behaviors were also reportedly obvious predictors of depression [13]. Longer time spent on social media was also shown to be positively related to depression symptoms [14]. The majority of studies repeatedly demonstrated that regular physical activities could promote psychological health and alleviate the levels of anxiety, depression and stress [15, 16]. Accordingly, to some extent, biological rhythm disorder should be regarded as a risk factor for psychological health problems.

In addition, people with biological rhythm disorder were more likely to have engaged in alcohol and cigarette use [17]. Furthermore, compared with continuing to smoke, smoking cessation was related to relieved depression and anxiety and an improvement in psychological health symptoms [18]. Adolescence is a susceptible period for the initiation of substance use, which occurs in the context of normative changes in reward circuitry, behavior, emotion, sleep and other risky behaviors. Owing to sleep deprivation and circadian misalignment, the developmental changes in the neural circuitry controlling reward function seem to be altered, which likely results in susceptibility to substance use [19]. Thus, keeping a normal biological rhythm, to some extent, can significantly reduce the use of tobacco and alcohol.

Moreover, cigarette smoking and alcohol drinking, which start in or are observed throughout adolescence [20], affect the status of psychological health and have been equally acknowledged [21, 22]. Several researchers have also reported more negative psychological health outcomes among individuals who smoke and consume alcohol frequently [21–23]. For example, Velten et al., in a representative population survey, indicated that smokers and people who drink excessively were more likely to experience depression, anxiety and lower life satisfaction than nonsmokers and moderate drinkers. They also reported that smoking and alcohol consumption may be individually predictive of psychological health status [24].

Reportedly, a larger social jetlag [25], more severe depression, and more frequent substance use [26] were observed in adolescents with evening preferences. Little, however, is known about the associations between biological rhythm and psychological health differentiated by smoking and drinking status. The research hypothesis arose that smoking and drinking worsen the correlations between biological rhythm disorder and psychological health problems. In view of the lack of research on gender differences, we did not make any a priori hypotheses regarding the moderating effects. To test these assumptions, we analyzed the relationships between biological rhythm and the multiple domains of psychological health (emotion disorders, behavioral problems and social adaptation symptoms) among adolescents, while we further explored whether smoking and drinking worsen the correlation and gender difference of the moderating effects in the study.

## Methods

### Participants

Three cities, including Shenzhen, Nanchang and Shenyang in China were chosen conveniently for this study. A cluster sampling method was conducted to select the study sample. First, we respectively extracted four large middle schools including junior and senior high schools and all participants were from grades 7 to 12, from each of the above three cities. Second, we randomly choose five classes for each grade in each chosen school and all students in selected classes were included in the survey, and about 2 700 students were chosen in each targeted region. Individuals with a family history of mental illness and being unwilling to participate in the survey were not included in the investigation. Finally, a total of 8,082 middle school students completed anonymous questionnaires in a classroom setting. Ninety-six incomplete questionnaires were excluded. The effective response rate was 98.8%. After excluding invalid questionnaires, including that the students were not at school on the investigation day, the students or their parents/caregivers were not reluctant to fill out the investigation questionnaire, and the students responded the questionnaires with an incomplete manner or dishonestly answered questionnaires or with logical errors, 7,986 adolescents, the range of the age from 8 to 23 years old, completed a standardized questionnaire finally, including 3,866 (48.4%) boys and 4,120 (51.6%) girls. In the study, only 0.1% of participants were less than 10-year-old who may have skipped a grade because of superior grades. Our study was approved and reviewed by the Ethics Committee of Anhui Medical University (Ethical No. 20,200,965). Our survey data were collected from October to December 2019, and all participants, parents and surveyed schools signed informed consent for inclusion before the administration of the survey.

### Measures

#### Sociodemographic data

In the present study, sociodemographic data for each participant were collected, including gender (boys/girls), age, registered residence (rural/urban), only child (yes/no), paternal and maternal education (< 12 years/≥ 12 years), and self-reported family economy (good/general/bad). In China, less than 12 years of schooling means no high school graduation.

### Psychological health

Mental health was evaluated using the Chinese version of the fifteen-item Brief Instrument on Psychological Health of Youths (BIOPHY-15) [27]. The questionnaire was divided into the following three dimensions to assess mental health in the past month: emotional symptoms, behavior problems and social adaptation symptoms. Emotional symptoms were measured by 7 items, e.g., “You often lose interest in things” and “You often blame yourself”. Behavior problems in the past 30 days were assessed with 4-item short statements, e.g., “You often argue with other people” and “You often lose control of your temper”. The questionnaire comprises four items related to social adaptation symptoms, e.g., “You are often reluctant to ask for help when in trouble”. Each item had six possible answers: 1= “lasting ≥ 3 months”, 2= “lasting ≥ 2 months”, 3= “lasting ≥ 1 months”, 4= “lasting ≥ 2 week”, 5= “lasting ≥ 1 week”, 6= “none or lasting < 1 week.” The psychological symptoms were assessed by calculating the total score of all items; the total score ranges from 15 to 90; a lower score on the questionnaire implied more severe symptoms [27]. The skewness of BIOPHY-15 was − 1.093 ± 0.027, the kurtosis was 0.405 ± 0.055. The total Cronbach’s α coefficient of the psychological symptoms self-rating scale was 0.928, and the Cronbach’s α coefficients of the three dimensions (emotional symptoms, behavior problems and social adaptation symptoms) were 0.885, 0.815, and 0.777, respectively in the study.

### Biological rhythm

Biological rhythm status was assessed using the Self-Rating of Biological Rhythm Disorder for Adolescents (SBRDA) [28]. The SBRDA consists of 29 items, reflecting four dimensions: sleep, digital media use, eating habits, and activities. Participants were required to think about their true biological rhythm status during the past 30 days. Sleep was evaluated by six items, e.g., “You get up an hour and a half later on weekends” and “You need an alarm clock or someone else to wake up in the morning”. Digital media use was measured by eight items, e.g., “You feel irritable or frustrated when you haven’t looked at your digital media for a long time”. Eating habits were assessed with eight-item, e.g., “You can’t keep eating regular amounts”. Activities were measured by seven-item, e.g., “You can’t keep pace with your family in your daily life”. Each item was rated on a five-point Likert scale, ranging from 1 = “completely untrue”, 2 = “basically untrue,” 3 = “somewhat true,” 4 = “basically true,” to 5 = “completely true.” The composite SBRDA score ranged from 29 to 145, corresponding to the levels from none to extreme; the higher the score on the questionnaire, the deeper the degree of biological rhythm disorder. The skewness of SBRDA was 0.351 ± 0.027, the kurtosis was 0.283 ± 0.055. The internal consistency of the SBRDA and each dimension, including sleep, digital, eating habits, and activities, in this study were 0.950, 0.834, 0.904, 0.893, and 0.817, respectively, which had also been confirmed by a sample containing approximately 10,000 adolescents [28].

### Smoking

Smoking among adolescents was evaluated based on the Youth Risk Behavior Survey System (YRBSS) [29]. The one question, “During the past month, how many days have you smoked cigarettes?” including four options (0 day, one to nine days, ten to nineteen days and twenty to thirty days), was used to measure the status of smoking.

### Drinking

Drinking was assessed based on the Youth Risk Behavior Survey System (YRBSS) [30]. “During the past a month, how many days did you drink a glass of wine (a glass of wine is equivalent to half a bottle of beer/a can of beer, a small cup of white wine, a glass of wine or rice wine)?” The question had four selection categories (0 day, one to nine days, ten to nineteen days, more than twenty days).

The validity of self-reported data on behaviors in adolescence that are relevant to smoking and drinking has been assessed [31, 32].

### Statistical analysis

All statistical analyses in this study were conducted by SPSS version 23.0 (SPSS, Chicago, IL, USA). We assessed the sociodemographic characteristics of the sample using descriptive statistics. We conducted a chi-square test to compare the gender differences among different sociodemographic characteristics. Spearman correlation analysis was performed to test the correlations between smoking, drinking, biological rhythm, and mental health among Chinese adolescents and to examine gender differences. In SPSS PROCESS, the interacting effects (biological rhythm×smoking and biological rhythm×drinking) were calculated automatically on the software to explore whether smoking and drinking moderated the associations between biological rhythm and mental health among adolescents. and they also produced the proportions of the variances explained by the moderating effects of smoking and drinking (*R*^2^ and *F* increased due to interaction). We adjusted the effects of sociodemographic characteristics (registered residence, only child status, and self-reported family economy) in the moderation models and compared the moderating effects between boys and girls, and the bootstrapping analysis with 5000 replicates was performed to determine the significance of the moderation effects. All *P* values were 2 tailed, and *P* < 0.05 implied that the moderating effects were statistically significant.

## Results

### Characteristics of participants

Of the 7,986 adolescents, the mean age was 14.7 ± 2.0 years, the average age of the boys was 14.7 ± 2.0, and that of the girls was 14.7 ± 1.9. In total, 31.9% of the participants’ registered residences were rural, 34.7% of the participants were only children, and all there were gender differences (*P* < 0.05 and *P* < 0.001, respectively). The self-reported family economic status fell into the following groups: 12.9% were bad economy families, 66.2% were general economy families, and 20.9% were good economy families. Compared with girls, boys had significantly higher child trauma scores, a lower level of biological rhythm and fewer suicidal behaviors (including suicidal ideation, suicidal plan, and suicidal attempt) (*P* < 0.001). The details of gender differences can be found in Table 1.

**Table 1:** Descriptive statistics for variables

Variables	Total *N* = 7 986(%)	Boys N = 3 866(%)	Girls N = 4 120(%)	*P* Value
Age (Mean ± SD)	14.7 ± 2.0	14.7 ± 2.0	14.7 ± 1.9	0.28
Registered residence				
Rural	2 547 (31.9)	1 188 (46.6)	1 359 (53.4)	< 0.05
Urban	5 439 (68.1)	2 678 (49.2)	2 761 (50.8)	
Only child				< 0.001
Yes	2 769 (34.7)	1 494 (54.0)	1 275 (46.0)	
No	5 217 (65.3)	2 372 (45.5)	2 845 (54.5)	
Paternal education				0.93
< 12 years	3 427 (42.9)	1 661 (48.5)	1 766 (51.5)	
≥ 12 years	4 559 (57.1)	2 205 (48.4)	2 354 (51.6)	
Maternal education				0.87
< 12 years	3 891 (48.7)	1 880 (48.3)	2 011 (51.7)	
≥ 12 years	4 095 (51.3)	1 986 (48.5)	2 109 (51.5)	
Self-reported family economy				< 0.001
Bad	1 030 (12.9)	565 (54.9)	465 (45.1)	
General	5 284 (66.2)	2 448 (46.3)	2 836 (53.7)	
Good	1 672 (20.9)	853 (51.0)	819 (49.0)	
BR (Mean ± SD)	71.90 ± 18.59	69.99 ± 18.73	73.69 ± 18.29	< 0.001
Psychological health (Mean ± SD)	72.32 ± 18.15	73.07 ± 18.17	71.61 ± 18.10	< 0.001
Emotional disorder (Mean ± SD)	33.01 ± 9.61	33.56 ± 9.62	32.50 ± 9.58	< 0.001
Behaviour problem (Mean ± SD)	20.43 ± 5.12	20.58 ± 5.03	20.30 ± 5.20	< 0.05
Social adaptation (Mean ± SD)	18.87 ± 5.77	18.94 ± 5.79	18.81 ± 5.75	0.30
Smoking (Mean ± SD)	1.07 ± 0.40	1.10 ± 0.49	1.04 ± 0.31	< 0.001
Drinking (Mean ± SD)	1.14 ± 0.49	1.18 ± 0.54	1.11 ± 0.42	< 0.001

### Correlation between smoking, drinking and biological rhythm and psychological health

The results from Spearman correlation analysis showed significant correlations between biological rhythm and psychological health (*r* = -0.43, *P* < 0.001), emotional disorder (*r* = -0.40, *P* < 0.001), behavior problems (*r* = -0.39, *P* < 0.001), social adaptation (*r* = -0.37, *P* < 0.001), smoking (*r* = 0.11, *P* < 0.001), and drinking (*r* = 0.21, *P* < 0.001). Spearman correlation analyses also revealed a marked link between psychological health and smoking (*r* = -0.09, *P* < 0.001) and drinking (*r* = -0.16, *P* < 0.001) (see Table 2).

**Table 2:** Correlations among smoking, drinking, biological rhythm and psychological health in Chinese adolescents

Variable	1	2	3	4	5	6	7
Total							
1.biological rhythm	1						
2.psychological health	-0.43**	1					
3.emotional disorder	-0.40**	0.93**	1				
4.behaviour problem	-0.39**	0.78**	0.66**	1			
5.social adaptation	-0.37**	0.86**	0.68**	0.61**	1		
6.smoking	0.11**	-0.09**	-0.07**	-0.09**	-0.08**	1	
7.drinking	0.21**	-0.16**	-0.14**	-0.15**	-0.15**	0.28**	1
Boys							
1.biological rhythm	1						
2.psychological health	-0.40**	1					
3.emotional disorder	-0.36**	0.93**	1				
4.behaviour problem	-0.34**	0.79**	0.66**	1			
5.social adaptation	-0.36**	0.86**	0.68**	0.62**	1		
6.smoking	0.14**	-0.10**	-0.09**	-0.09**	-0.08**	1	
7.drinking	0.23**	-0.17**	-0.15**	-0.15**	-0.15**	0.29**	1
Girls							
1.biological rhythm	1						
2.psychological health	-0.46**	1					
3.emotional disorder	-0.43**	0.94**	1				
4.behaviour problem	-0.42**	0.78**	0.66**	1			
5.social adaptation	-0.38**	0.85**	0.68**	0.60**	1		
6.smoking	0.09**	-0.09**	-0.07**	-0.10**	-0.08**	1	
7.drinking	0.21**	-0.17**	-0.15**	-0.16**	-0.15**	0.25**	1

Note: * *P* < 0.05; ** *P* < 0.001

### Moderation effect of smoking and drinking between biological rhythm and psychological health

In Tables 3 and 4, we observed that smoking (*B*=-3.08, *p* < 0.05) and drinking (*B*=-4.42, *p* < 0.05) separately negatively predicted psychological health status, while the above negative prediction effects were significant in boys (*B*=-2.46, *p* < 0.05; *B*=-3.99, *p* < 0.05) and girls (*B*=-4.67, *p* < 0.05; *B*=-5.35, *p* < 0.05). Additionally, smoking (*B* = 0.05, *p* < 0.05) and drinking (*B* = 0.06, *p* < 0.001) respectively accelerated the association between biological rhythm and psychological health in the total sample. Classified by genders, among girls, smoking accelerated the association between biological rhythm and psychological health (*B* = 0.09, *p* < 0.05), and drinking also accelerated the association between biological rhythm and psychological health (*B* = 0.12, *p* < 0.05). Among boys, smoking (*B* = 0.03, *p >* 0.05) and drinking (*B* = 0.02, *p >* 0.05) did not accelerated respectively the association between biological rhythm and psychological health.

**Table 3:** Moderating effect of smoking between biological rhythm and psychological health

Variables	*B*	*95% CI*	*SE*	*t*
Total ^a^				
biological rhythm	-0.39	(-0.41, -0.37)	0.01	-38.15**
smoking	-3.08	(-4.11, -2.04)	0.53	-5.83**
biological rhythm×smoking	0.05	(0.02, 0.09)	0.02	2.90*
*R*^2^, *F*	*R*^2^ = 0.17, *F =* 240.89**			
*ΔR*^2^, *ΔF*	*ΔR*^2^ = 0.001, *ΔF =* 8.26*			
Boys ^b^				
biological rhythm	-0.34	(-0.37, -0.31)	0.01	-22.75**
smoking	-2.46	(-3.74, -1.18)	0.65	-3.77**
biological rhythm×smoking	0.03	(-0.02, 0.07)	0.02	1.28
*R*^2^, *F*	*R*^2^ = 0.14, *F =* 283.89**			
*ΔR*^2^, *ΔF*	*ΔR*^2^ = 0.0004, *ΔF =* 1.63			
Girls ^b^				
biological rhythm	-0.44	(-0.46, -0.41)	0.01	-31.36**
smoking	-4.67	(-6.54, -2.80)	0.95	-4.90**
biological rhythm×smoking	0.09	(0.02, 0.15)	0.03	2.63*
*R*^2^, *F*	*R*^2^ = 0.21, *F =* 259.37**			
*ΔR*^2^, *ΔF*	*ΔR*^2^ = 0.001, *ΔF =* 6.92*			

Note. *p < 0.05, **p < 0.001; aAdjusted for gender, registered residence, only child status, self-reported family economy; bAdjusted for registered residence, only child status, and self-reported family economy

**Table 4:** Moderating effect of drinking between biological rhythm and psychological health

Variables	*B*	*95% CI*	*SE*	*t*
Total ^a^				
biological rhythm	-0.38	(-0.40, -0.35)	0.01	-36.66**
drinking	-4.42	(-5.28, -3.57)	0.44	-10.16**
biological rhythm×drinking	0.06	(0.03, 0.09)	0.02	3.50**
*R*^2^, *F*	*R*^2^ = 0.18, *F =* 253.11**			
*ΔR*^2^, *ΔF*	*ΔR*^2^ = 0.001, *ΔF =* 12.22**			
Boys ^b^				
biological rhythm	-0.32	(-0.35, -0.29)	0.01	-21.71**
drinking	-3.99	(-5.10, -2.88)	0.57	-7.03**
biological rhythm×drinking	0.02	(-0.03, 0.06)	0.02	0.78
*R*^2^, *F*	*R*^2^ = 0.15, *F =* 114.08**			
*ΔR*^2^, *ΔF*	*ΔR*^2^ = 0.0001, *ΔF =* 0.60			
Girls ^b^				
biological rhythm	-0.43	(-0.45, -0.40)	0.01	-30.35**
drinking	-5.35	(-6.73, -3.97)	0.70	-7.61**
biological rhythm×drinking	0.12	(0.06, 0.17)	0.03	4.40**
*R*^2^, *F*	*R*^2^ = 0.22, *F =* 188.96**			
*ΔR*^2^, *ΔF*	*ΔR*^2^ = 0.004, *ΔF =* 19.41**			

Note. *p < 0.05, **p < 0.001; aAdjusted for gender, registered residence, only child status, self-reported family economy; bAdjusted for registered residence, only child status, and self-reported family economy

## Discussion

The present study explored the correlations among biological rhythm, smoking, drinking and psychological health and assessed the moderating effects of smoking and drinking on the correlation between biological rhythm and psychological health while gender differences were taken into account. The study showed that significant associations existed between biological rhythm, smoking, drinking and psychological health in adolescents. Additionally, smoking and drinking aggravated the negative influences of biological rhythm disorder on psychological health, which varied by gender. In the total sample and the girls, smoking will accelerate the effect of biological rhythm disorder on psychological health, such as drinking. However, there were no moderating effects of smoking or drinking in boys.

### Associations between Biological Rhythm, Smoking, drinking, and Psychological Health

Biological rhythm disorder was observed to be negatively associated with psychological health, which was similarly reported in other studies [33, 34]. Previous research has demonstrated a consistent correlation between biological rhythm disorder and a higher prevalence of psychological problems among adolescents [35]. Additionally, adolescents with a more dysfunctional biological rhythm are more likely to engage in cigarette use and alcohol consumption, which could contribute to the biological rhythm disruption that increases their susceptibility to substance use [19].

As has been acknowledged in recent studies, we found a positive correlation between substance abuse and psychological health. A self-reported investigation focusing on college students in Spain also showed that students with substance use were more likely to suffer from depression and anxiety symptoms [36]. If giving up smoking and drinking, positive outcomes will appear with fewer psychological symptoms.

### The moderating roles of smoking and drinking between biological rhythm and psychological health

The present study shows that smoking and drinking worsen the correlations between biological rhythm disorder and psychological health in adolescents. Tobacco users exhibit depression, agitation and anxiety, which are attributed to the promoting effects of tobacco on neuroadaptations in nicotinic pathways in the brain, which causes poor psychological health [37]. Psychological, behavioral or psychological responses triggered by alcohol consumption may interfere with the individual’s adaptability, which contributes to psychological problems [38].

People suffering from biological rhythm disorders smoke and drink more than normal persons [17]. Additionally, reportedly, individuals with biological rhythm disorders were associated with greater nicotine use and dependence and greater alcohol consumption [39, 40]. It seems that poor psychological health among individuals with biological rhythm disorder is exacerbated by smoking and drinking on account of the synergistic effects of smoking, drinking and biological rhythm disruption on psychological health.

Another notable finding is that smoking and drinking, as moderating variables, have gender-specific effects on the relationship between biological rhythm disorder and psychological disorder. These moderating roles significantly accelerate the effect of biological rhythm disorder on psychological health only in girls, and stronger associations between biological rhythm, smoking, drinking and psychological health among girls are displayed. The reasons for these differences may be the narrowing gender gap in the prevalence of substance abuse, which is characterized by an increasing representation of girls [41]. Moreover, girls exhibit greater psychological vulnerability than boys [42], consequently resulting in a greater risk to psychological health. and girls are liable to exhibit biological rhythm disorder and a greater cortisol awakening response [43], which may be a symbol of psychological stress [44]. More importantly, substance use could bring about or exacerbate existing psychological health problems [45, 46]. Therefore, girls, but not boys, experiencing the disorder of biological rhythm, especially those who engage in smoking or drinking alcohol, are associated with poor psychological symptoms.

### Limitations and Strengths

There are several limitations in the present study. First, owing to the cross-sectional study design, the converse associations and causal associations between biological rhythm disorder and psychological health cannot be inferred. Second, data were based on self-report, which was prone to recall bias. Third, smoking and drinking, classified only in a binary way in the study, did not take more detailed characteristics or joint effects into account. Both smoking and drinking had very small but statistically significant moderating effects on biological rhythm disorder and psychological health. The adverse effects of smoking and drinking have been thoroughly established in previous research, but the attention may be more played to reduce the prevalence of smoking and drinking among girls with biological rhythm disorder to promote psychological health in the future. Psychological health, affected by complicated factors, may also be influenced by health care resources and utilization, which may lead to the small moderating effects of substance use on biological rhythm disorder and psychological health. Therefore, further research is needed to clarify the moderating effects. Nevertheless, to our knowledge, this is the first study to detect the moderating roles of smoking and drinking on the correlation between biological rhythm disorder and psychological health and gender differences in Chinese adolescents, which covers urban and rural areas.

### Implications for practice and research

The present research explores the potential relationships between biological rhythm disorder, smoking, drinking, and psychological health. In formulating the primary preventive measures concentrating on the psychological health of adolescents, especially girls, reducing tobacco and alcohol use or cessation treatment need to be taken into consideration by health care professionals to improve psychological symptoms with biological rhythm disorder. More longitudinal studies are needed to confirm these findings in the future. More efforts are needed to explore why smoking and drinking effects accelerate the relationships between biological rhythm disorder and psychological health problems. Further research should consider the joint effect of smoking and drinking and the dose responses of tobacco and alcohol consumption to obtain a fuller picture of the impacts of smoking and drinking on the relationship.

## Conclusions

This study filled a gap in knowledge regarding the effects of smoking and drinking on the correlation between biological rhythm disorder and psychological health among adolescents. Remarkable moderating effects of smoking and drinking on the correlation were observed. However, we only observed moderating effects in girls but not in boys. Further rigorous studies are needed to explore the mechanism of gender differences in moderating effects.

### Electronic supplementary material

Below is the link to the electronic supplementary material.

Supplementary material 1: Supplement Figure 1 and Supplement Table 1

## Data Availability

Owing to the appropriate protection of participants’ personal information, the original datasets used and analyzed in the current study are not publicly available, but are available from the corresponding author on reasonable request.
