# Supplementary material for: Moderating effects of smoking and drinking on the relationship between biological rhythm and psychological health and gender differences among adolescents
Source: BMC Psychiatry. 2023 Oct 10;23:731. doi: 10.1186/s12888-023-05253-2 (PMC10566120; doi:10.1186/s12888-023-05253-2)
Supplement: Supplementary file 1 — Supplementary material 1: Supplement Figure 1 and Supplement Table 1 [file 12888_2023_5253_MOESM1_ESM.docx]

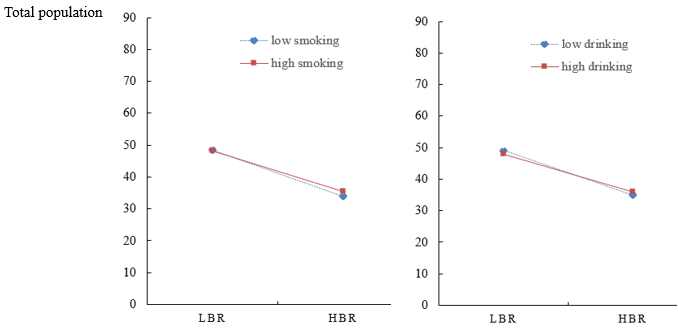


psychological health


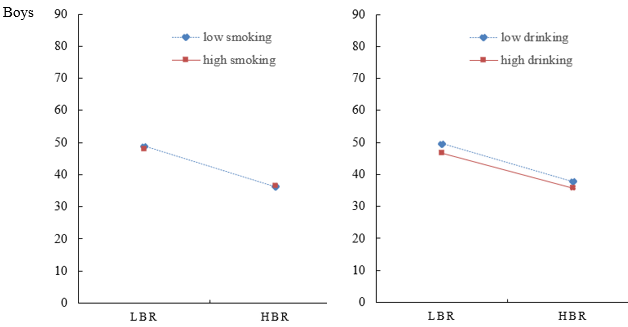


psychological health


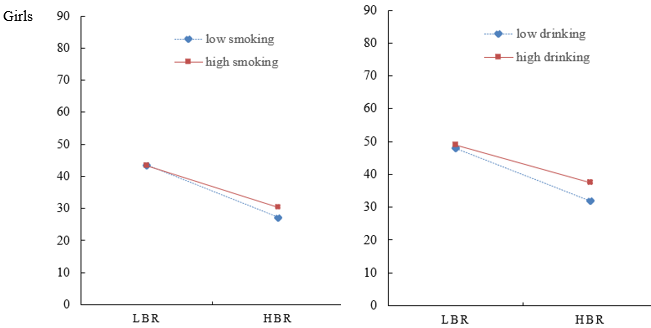


psychological health

Supplement figure 1 Significant interaction of smoking/drinking and biological rhythm on psychological health.

Supplement table 1 Three-way interaction between biological rhythm, smoking/drinking and gender.

| Variables | Smoking | | | |  | Drinking | | | |
| --- | --- | --- | --- | --- | --- | --- | --- | --- | --- |
|  | *B* | *95% CI* | *SE* | *t* |  | *B* | *95% CI* | *SE* | *t* |
| Biological rhythm | -0.22 | (-0.35, -0.08) | 0.07 | -3.12* |  | -0.14 | (-0.28, -0.001) | 0.07 | -1.97* |
| Moderator | 2.06 | (-7.53, 11.64) | 4.89 | 0.42 |  | 3.31 | (-5.52, 12.13) | 4.50 | 0.73 |
| Gender | 13.33 | (5.23, 21.44) | 4.13 | 3.23* |  | 16.12 | (8.77, 23.48) | 3.75 | 4.30** |
| Int_1 | -0.03 | (-0.14, 0.08) | 0.05 | -0.54 |  | -0.08 | (-0.18, 0.02) | 0.05 | -1.49 |
| Int_2 | -0.15 | (-0.25, -0.06) | 0.05 | -3.20* |  | -0.21 | (-0.30, -0.12) | 0.05 | -4.52** |
| Int_3 | -6.59 | (-13.66, 0.49) | 3.61 | -1.82 |  | -8.58 | (-14.61, -2.54) | 3.08 | -2.79* |
| Int_4 | 0.06 | (-0.02, 0.14) | 0.04 | 1.47 |  | 0.10 | (0.03, 0.17) | 0.04 | 2.76* |
| *R*^2^, *F* | *R*^2^=0.18, *F*=171.758** | | | |  | *R*^2^=0.18, *F*=171.758** | | | |
| *ΔR*^2^, *ΔF* | *ΔR*^2^=0.0002, *ΔF*=2.15 | | | |  | *ΔR*^2^=0.001, *ΔF*=7.61* | | | |

Note. **p* < .05, ***p* < .001; aAdjusted for gender, registered residence, only child status, self-reported family economy; bAdjusted for registered residence, only child status, and self-reported family economy.

Int_1: biological rhythm × smoking and biological rhythm × drinking.

Int_2: biological rhythm × gender.

Int_3: smoking × gender and drinking × gender.

Int_4: biological rhythm × smoking × gender and biological rhythm × drinking × gender.
